# Supplementary material for: Sequences at gene segment termini inclusive of untranslated regions and partial open reading frames play a critical role in mammalian orthoreovirus S gene packaging
Source: PLoS Pathog. 2024 Feb 23;20(2):e1012037. doi: 10.1371/journal.ppat.1012037 (PMC10917250; doi:10.1371/journal.ppat.1012037)
Supplement: Attachment — Submitted filename: Dhar and Miller 2023 Response to review_Final.docx [file ppat.1012037.s001.docx]

**General response to review:**

**We thank the editors for allowing us to submit a revised manuscript and the reviewers for suggesting changes to improve our findings. We appreciate the time and effort taken to provide feedback and are grateful that the reviewers have acknowledged the novelty of our findings and appreciated the challenging nature of our project. We have carefully considered all suggestions and addressed them to the best of our abilities. We feel strongly that the findings of our manuscript have been strengthened further after addressing all comments and concerns. As suggested by the reviewers, we have added experiments to address their concerns, which has resulted in an addition of figures and changes of figure numbers as mentioned in the table below.**

**In the following pages of this document, we have separated all the comments and concerns raised by the reviewers and addressed each one individually. A compare copy/redlined version of our original manuscript highlighting the changes made and a corresponding clean version incorporating the changes have been included in our re-submission (Note: all line numbers mentioned within this document correspond to the line numbers of the clean version of our submission).**

**Table 1: Figure numbers of the original draft and changes made in the new submission**

| ***Figure number*** | |
| --- | --- |
| ***Original draft*** | ***This submission*** |
| **Figure 1 A and B** | **Figure 1 A and B** |
| **Figure 2 A, B and C** | **Figure 2 A, B and C** |
| **Figure 2 D, E, F, G, H** | **Figure 3A, B, C, D, E, F, G** |
|  | **New figures 3H, I and J added** |
| **Figure 3 A and B** | **Figure 4 A and B** |
| **Figure 4 A-E** | **Figure 5 A-E** |
| **Figure 5 A-E** | **Figure 6 A-E** |
| **Figure 6 A-C** | **Figure 7 A-C** |
| **Figure 7 A-F** | **Figure 8 A-F** |
| **Figure 8 A-E** | **Figure 9 A-E** |
| **Figure 9 A and B** | **Figure 10 A and B** |

Reviewer #1:

Many questions remain regarding how multi-segmented dsRNA viruses faithfully package the correct assortment of segments into each virus particle. For ten-segmented reovirus, the 5' and 3' termini of RNA segments are predicted to fold into panhandle structures. Previous studies have shown that sequences encompassing the 5' and 3' untranslated regions (UTRs) and extending into the ORF are sufficient for segment packaging, including the packaging of foreign sequences. Systems used to make these findings often involved the use of helper viruses or duplicated sequences, which in some cases could confound interpretation of the results. In the current study, Dhar et al. modified the reovirus plasmid-based reverse genetics system to identify RNA sequences required for packaging (inclusive of segment assortment, assembly, and genome replication) through new virus recovery. Their approaches include the use of wobble/block replacement (W/BR) codon usage alterations and plasmid-based 'transcomplementation' of viral proteins encoded by altered segments. Using these approaches, they validated previous findings suggesting that the N termini of segments, including 5' and 3' untranslated regions (UTRs) and sequences extending into the ORF, are important mediators of reovirus segment packaging. They defined minimal nucleotide sequences sufficient for packaging the S1 segment. Using WB/R and segment incorporation assays, the authors provide evidence that internal segment sequences contribute minimally to RNA packaging. Using RNA structure prediction, the authors identify a six-nucleotide sequence in the 3' UTR, which is predicted form the loop portion of a stem loop structure, as being conserved across prototype MRV strains. They were unable to recover viable viruses following mutation of these residues, even with transcomplemetation, and conclude that the residues are required for packaging. By engineering segments that encode point mutations in the panhandle that are predicted to disrupt RNA secondary structure or sequence alone, the authors provide evidence that structure rather than sequence is important for segment packaging.

The assay systems presented are new and represent improvements over previous reovirus packaging assays, as they lack the confounders of helper virus or duplicated sequences. One caveat is that, as with previous packaging assay systems, the current system provides little insight into the replication step(s) at which packaging is impaired for viruses that fail to rescue or those that have reduced rescue titers. However, in experiments in which virus is not recovered even in the presence of transcomplemetation, it is now evident that the lack of rescue is not due to a lack of expression of the encoded protein. Some knowledge gained through the study validates prior findings, but several of the current findings are new. Specifically, minimal packaging signals were identified for the reovirus S1 segment, and new insights were made into potential roles of structure versus sequence and a conserved RNA element in the panhandle in packaging. The authors' conclusions are largely supported by their experimental findings. However, it would inspire even more confidence if conclusions about packaging efficiency were based on more than titers from reverse genetics experiments alone. Although translation of proteins encoded by altered reverse genetic plasmids was compared following transfection of BHK cells, it is possible that they will vary in the presence of the other viral transcripts. Overall, the current work contributes new methods to study viral packaging and new findings made using the assays. These findings make inroads into challenging questions related to the mysteries of RNA segment assortment and new virus assembly that have long challenged the dsRNA virus field.

**Concerns:**

**1(a)**. The consistency of rescue titers from experiment to experiment, combined with measures of protein expression and transcript levels from reverse genetics plasmids separately transfected in BHK cells, provides confidence that reverse genetics recovery titer is correlated with packaging efficiency rather than just indicating that viable virus recovery is possible. However, whether these factors are directly related is not entirely clear, as other factors could influence the outcome of rescue experiments. Quantitation of replication kinetics for recovered viruses with altered segments relative to those of the parental viruses, at least for those viruses that do not require transcomplementation, would provide an important comparison. This comparison would involve multiple rounds of amplification. It is likely that the same is true following a five-day reverse genetics transfection. However, in the case of a replication assay, an equal infectious dose would be used as the experimental input. In the case of a reverse genetics experiment, a substantial delay in initial virus recovery for any number of reasons might be amplified.

**Response: We acknowledge the reviewer's concern that in our original manuscript, we did not delineate whether the defect in the lack of viral recovery for certain mutants in the RG assay is due to significant defects in the packaging process of progeny virions (virions not produced or produced at lowered efficiency from infected cells) or defects in the replication kinetics of the virus (progeny virions are produced but are unable to infect the next round of cells). The reviewer suggests that by doing a time course replication assay with an equal infectious dose, it would provide some differentiation on whether the defect is in infectivity of mutant virions or later steps in replication. We agree with the reviewer, and have now included a multistep replication assay in L929 cells, where we infected L929 cells with 18/S1/40 (the only recovered S gene UTR-only virus), 200/S1/200, and S1 WT viruses and compared their replication over 72 hours to measure virus growth over time. The results from this assay have been added in Figure 3I, and clearly demonstrate that even when equivalent numbers of plaque forming units are added at time=0, the 18/S1/40 virus fails to efficiently grow over time relative to 200/S1/200 and WT S1. This defect in growth is also apparent in the small plaque phenotype from the original manuscript shown in Fig 2C. In thinking about this question, we realized there was a second experiment we could perform that more directly assesses whether packaging or virus infectivity (entry, transcription, and translation) is impacted in this mutant. In this assay we infected L929 cells at the same MOI, performed immunofluorescence using antibodies against a viral non-structural protein, and compared the number of infected cells in 18/S1/40 relative to S1 WT and 200/S1/200. The results from this assay indicate strongly that there is no defect in the early stages of infection (entry, transcription, translation) of the S1 UTR mutant, further implicating a late step such as packaging (assortment, assembly, replication) as being impaired. These results have also been added to the revised manuscript in Figure 3J. Finally, we have added an acknowledgement in the Discussion section that although we made substantial effort to rule out other steps in the virus life cycle that may be impacted by these mutations (such as measuring RNA and protein expression levels, providing the mutated protein in trans in the complementation assays, and not mutating the conserved 5’ and 3’ nt likely to be involved in RdRp binding and replication in any of our constructs), “the possibility still remains that mutants that we were unable to recover in our plaque assays may produce non-infectious virus with defects in processes needed to infect the next round of cells.” (Lines 589-591) ).**

**1(b).** Protein expression levels in BHK cells for altered segment constructs all were measured in the absence of expression of other viral transcripts/proteins. It is possible that expression levels differ for altered transcripts during reverse genetics experiments when all other viral transcripts are present in the cell and available to compete. It appears that much larger amounts of plasmid (10 µg) were transfected for protein expression experiments than for reverse genetics experiments (0.4 µg). If it is it possible to co-transfect the other RG plasmids and still detect expression of the protein of interest (e.g., 1-2 µg of each plasmid), then these experiments should be done to determine whether the presence of the other viral transcripts/proteins affects protein levels for altered constructs.

**Response:** **The reasoning behind adding much higher amounts of plasmids for transfection to measure protein expression was to attempt to counteract poor sensitivity/specificity of some of the σ antibodies at our disposal. We tried multiple different σ antibodies using increasing concentrations of S plasmids and had the best luck with those shown in the manuscript using 10ug of plasmid transfection. Immunofluorescence assays with lower amounts of plasmids (1ug) were also performed (data not shown in the manuscript), and we did not detect significant differences in σ protein expression between WT and the mutant constructs in most cases. However, as immunofluorescence assays are not the best quantitative assay to measure protein expression levels, we decided to only provide the results from our immunoblot assays in the manuscript.**

**Nonetheless, we agree with the reviewer that the absence of expression of the other viral proteins, some of which have been implicated in regulation of viral protein translation, may impact expression of the proteins we are measuring. To address this concern, we have repeated protein expression assays for a subset of our mutant plasmids for which we had the best antibodies (S1 and S3) in the presence of all the other RG assay plasmids. Our results from these experiments show no significant change in the expression patterns of WT and mutant σ1 and σNS compared to when they were expressed alone in higher amounts. The results from these experiments have been added in Fig 3H in the revised manuscript.**

1(c) Line 396-407: It is unclear why different RNA structure prediction algorithms were used to predict structures for S1 gene sequences from the three major MRV serotypes or a single S1 segment. Details of alignment strategies are not described in Materials and Methods. Was any setting other than the default applied?

**Response: We thank the reviewer for requesting further clarification.** **Two different RNA structure prediction algorithms (Mfold and Multilign) were used, where Mfold predicted single-sequence structures while Multilign was used for consensus RNA folding of multiple homologous sequences, particularly for the analysis of the panhandle structure.The program Multilign (1) is optimized for predicting global consensus structures of RNAs that have homologous sequences and structures. It uses dynamic programming to optimize both the secondary structure and the alignment. It optimizes an alignment score and a consensus minimum free energy simultaneously** **and can be used to find structural homology even when sequences are quite distant. We have added this clarification statement in the Results section (Lines 443-451).**

1(d) Line 488-490: RNA secondary structure and its disruption through engineered mutations still are only predicted computationally, though the current findings do provide additional support for the predictions. Perhaps phrasing could be altered slightly to indicate that the structures still are predictions.

**Response: We have now changed the line to "An RNA secondary structure was predicted computationally to exist by base pairing of the terminal ends of the MRV genome segments. Our data lend experimental support that this predicted secondary structure may play an important role in MRV genome packaging" (Lines 538-541)**

1(e) Line 624-639: While general construction of wobble mutated S gene segment-encoding plasmids is described, their sequences are not reported or deposited. Thus, it is not possible for another group to replicate the study.

**Response: We sincerely thank the reviewer for pointing this out and now we have added a file in our data share file (Figshare: “Packaging of mammalian orthoreovirus S gene segments”) containing sequences of plasmids that have been used In this manuscript. This data share file is available publicly through the Iowa State University library for everyone to view and download electronically and will be referenced in “Data Availability” section of the manuscript should it be accepted for publication.**

1(f) Line 711-723: Some details of the segment incorporation assay in which S1 terminal sequences were tested for the potential to mediate packaging of NanoLuc are unclear.

- Were equal volumes of lysate from each experiment used to infect the next round of cells (most likely, yes)?

**Response: Yes. Equal volumes of lysate were used for infecting the next round of cells. We have now changed the sentence to "Equal volumes of DNase I treated samples were then co-infected with WT T1L into L929 cells plated in 12 well dishes at 2x10^5^ cells per well"(Lines 785-787).**

- Please describe the control treatment with pNL3.1 to which infections were compared in Fig. 6B. Was this simply a plasmid transfection conducted alongside the infection?

**Response: In Fig 6C (now Fig 7C), each of the S1/NL/S1 chimeric plasmids were transfected individually into BHK-T7 cells and measurement of NL expression was compared to the positive control (pNL3.1) (also mentioned in Lines 381-385 of the original submission)***.* **This was done** **to address whether any differences in chimeric NL virus rescue and corresponding NL expression are due to any inherent defect of NL protein expression. We have added this clarification statement to the manuscript (Lines 423-425).**

- Also, why were DNase I treated samples co-infected with WT T1L rather than just being added to L929 cells? Please very briefly describe the purpose of the co-infecting virus.

**Response:** **DNaseI treated samples were coinfected with WT T1L** **to support additional rounds of NanoLuc/S1 gene packaging and corresponding infection for better detection of the NanoLuc signal from the infected cells. A clarification statement has been added to the manuscript in Line 787-789***.*

1 (g) Although translation of proteins encoded by altered reverse genetic plasmids was compared following transfection of BHK cells, it is possible that they will vary in the presence of the other viral transcripts.

**Response: See response to 1(b)**

1(h) The assay systems presented are new and represent improvements over previous reovirus packaging assays, as they lack the confounders of helper virus or duplicated sequences. One caveat is that, as with previous packaging assay systems, the current system provides little insight into the replication step(s) at which packaging is impaired for viruses that fail to rescue or those that have reduced rescue titers.

**Response: We acknowledge that the sequences that we have identified in our experiments do not narrow down which step in the packaging process is impacted. In the original draft, we have mentioned at multiple places that we are defining packaging as a combination of three processes (assembly, assortment, and replication/second strand synthesis). As an example, we wrote in the lines 126-130, "based on published data from MRV and other viruses with a dsRNA genome, we have hypothesized that signal sequences present at the terminal ends of each MRV gene segment inclusive of UTRs and partial ORFs guide the overall process of packaging (assortment, assembly, and replication). Therefore, the identified packaging signal may have a functional impact on any of the steps, including the possibility of impacting the onset of negative strand/second strand RNA synthesis”.**

**That being said, our results provide some indication that our designed mutations likely impact the RNA assortment step of packaging. Identifying the functional significance of the packaging sequences will require the development of a mammalian orthoreovirus (MRV) specific assortment assay, which is one of the ongoing focus areas in our laboratory (Lines 546-563).**

Reviewer #2

Using mammalian orthoreovirus (MLV), this manuscript by Dhar et al describes a clever approach for defining 5' and 3' terminal regions requiring nucleotide conservation for efficient virus rescue and replication. Specifically, they introduced wobble mutations into increase intergenic regions and monitored for loss of virus rescue and plaque size. The authors furthermore excluded that their modifications were merely reducing the levels of viral mRNAs and proteins expressed from the reverse genetics plasmids. While I cannot be entirely sure that the identified 3' and 5' sequences are all specifically essential for "packaging" as stated (explained further below), this study clearly defines the extremities of MLV that can tolerate nucleotide modifications. The researchers then applied their findings to incorporate a fully exogenous gene-encoding segment into progeny MLVs, albeit these progenies were replication defective as would be expected. Overall, the approach is clever and the experiments comprehensively done and described. The findings help define the nucleotide-sequence-dependent regions of MRV at 3'and 5' ends, but moreover will appeal to a more general audience for the approach.

**Concerns:**

2(a) I would suggest providing alternative interpretations to absence of infectious virus rescue that are still compatible with the results but not necessarily a direct reflection of packaging. As an example, if progeny virus particle packaged a genome but required nucleotide sequence or structure for onset of negative sense RNA synthesis etc. While assembly is most likely, I am not convinced it is the only RNA-dependent process between mRNA/protein expression from the reverse genetics plasmids (measured) to viruses capable of producing titer (measured).

**Response: We thank the reviewer for this suggestion, and we acknowledge that the functionality of the identified packaging sequences have not yet been fully determined. In particular, with known experimental approaches it is very difficult to separate the steps of assortment/assembly/replication, therefore, we stress multiple times in our manuscript that when we say “packaging” we are including assortment/assembly/replication. However, we believe in addressing concern 1(h) above in the revised manuscript, we have also made significant improvements to the manuscript related to this concern. See response 1(h).**

2(b) Line 104 – the statement that the specific infectivity of reovirus is <2 (particle/PFU) uses references that are older than many more-recent assessments that show a ratio of ~100-300 on average. As one example, see PMID: 22811534; but many studies find similar ranges. This still does not affect the major point of your argument, since random reassortment of 10 segments would give a much lower specific infectivity than 100-300.

**Response: We thank the reviewer for pointing this out, and now we have added two new references to clarify the specific infectivity of reovirus. We have removed the descriptive detail "<2" and changed the sentence to "Another line of evidence that suggests that MRV uses a selective packaging mechanism is that the ratio of total particles to infectious particles for MRV is low, indicating that the MRV packaging mechanism efficiently produces progeny virions with an entire complement of genes" (Lines 103-106)**

2 (c) For references in lines 110-118, it would help to indicate the virus these steps were discovered with as they may/may not directly apply to MLV and as written, it rather seems as if the references are providing proof that it is for MLV specifically.

**Response: Changed. The lines 114-117 now states, “Recent evidence from MRV serotype 3 and RV suggests that a single-layered particle (SLP) may form as an assembly intermediate and that the assortment complex is brought to the SLP by the RdRp (λ3, MRV; VP1,VPI), which is bound to each gene segment terminus".**

2(d) In lines 133-147 describing previous work on identifying packaging signals, it seems important to describe what the packaging signals were discovered to me, and why there was a need to further examine this question.

**Response: We thank the reviewer for seeking further clarification on this. We have added a sentence “ For example, these experiments indicated that the terminal 200 nts in S2, S4, M1 and L1 genes of the Type 3 Dearing serotype likely play a role in packaging” Lines 143-145, indicating the findings from the cited publications (2-6) in that paragraph. The following lines “However, these approaches were experimentally complex, relying on helper cell lines or the addition of duplicated sequences. Furthermore, in some cases these approaches could not rule out the possibility of wildtype (WT) sequences within the internal regions of the ORF contributing to the packaging process. Moreover, these approaches often did not identify minimal RNA sequences or structures required for the packaging process” (Lines145-150) explains our rationale of why there was a need to further examine the question.**

**Reviewer#3**

In this paper, Dhar et al define determinants in the mammalian reovirus S segments that control packaging efficiency. Using a clever strategy, the authors generate mutant viral gene segments such that the entire ORF is wobbled as much as possible. This strategy ensures that putative packaging signals are disrupted without changes in the amino acid sequence. Using this strategy, the authors systematically analyze the sequences in the viral genome segments necessary to allow packaging of the viral gene segments. With particular focus on the viral S1 gene segment, the authors identify the minimal sequence necessary. These sequences extend beyond the UTR regions. They also uncover a role for a previously unidentified panhandle structure and loop region in the UTR for packaging. Finally, they identify regions that are sufficient to allow packaging of a gene segment containing a trans gene using a complementation system. The experiments in this paper are clear, well presented and carefully controlled. I particularly appreciate the care taken to ensure that mutations introduced by the authors do not influence the other functions of the genome segment such as RNA synthesis and translation. The findings are new and exciting and will make an important new contribution to the field.

**Concerns:**

The paper can be improved in the following ways

3(a). Packaging is being measured by surrogate method - ie recovery of virus. It keeps open the possibility that packaging does indeed occur but the virus produced is non-infectious. The authors should consider alternate methods - such as qPCR from "virus fraction" to assess packaging. Perhaps showing that with one a few select mutants will be helpful.

**Response: We agree with this reviewer that this is a valid point, and one which has been raised by other reviewers. In this study, we specifically used this approach because it allowed us to reasonably quickly identify sequences that were necessary to produce replicating virus. We measured RNA and protein expression levels and performed transcomplementation assays to try to rule out any other possible reason for loss of virus replication, however, we did not specifically address the possibility that our lysates contained fully replicated virions that were just not infectious. We have now added a statement in the discussion to clarify that this remains a possibility (Lines 564-591). In addition, this concern is closely aligned to the concern that was raised by reviewer 1 in 1(h), and we have added replication and immunofluorescence assays to demonstrate that in the case of the S1 UTR-only virus, the virions are just as infectious as WT, and the defect appears to occur at a later step in the replication cycle (assortment, assembly and or replication). [See response to 1(h)].**

3(b). In the original paper on reovirus reverse genetics (Kobayashi et al 2007), it was shown that after transfection of T7 vectors expressing reovirus RNAs, very few cells are capable of producing virus. In other words, only a few received all 10 plasmids. Recovery of sufficient virus titer after reverse genetics required incubation over several days and the spread of virus to new cells that did not receive all 10 plasmids. Therefore, the experiments showing western blots for sigma1 expression are a little bit puzzling. One would expect very low level detection (if any) for viruses that are not recoverable. Yet, the expression seems reasonably high. It might be helpful to the reader if the authors demonstrate that this expression does not require spread of virus through the population - perhaps by blocking secondary rounds of infection through addition of entry inhibitors or neutralizing antisera.

**Response: We thank the reviewer for seeking further clarification on this. However, the RNA and protein expression levels that we have shown throughout the manuscript are from expression of our plasmid constructs transfected alone into cells, and not from RG assays. The reason we examined expression from plasmid constructs was twofold: 1) as the reviewer suggests, we were unable to detect viral proteins from RG assays because of low expression and/or poor antibody sensitivity/specificity and 2) to determine if the mutations that we were introducing had any impact on RNA/protein expression from the plasmid that might impact the RG assay results. In response to another reviewer concern, we have now added new data from a subset of our plasmids following coexpression with all of the other RG assay plasmids to also rule out any impact of other viral proteins on expression. See response to 1b.**

3(c) A direct measurement of packaging for select set of mutants would be helpful to ensure that it's truly a packaging defect.

**Response: We fully agree with the reviewer and strongly believe that an in vivo assay or set of assays is needed that separates assortment from replication. We currently have the capacity to measure replication using RT-qPCR on the negative strand and have done these experiments on some of the constructs in this manuscript (data not shown), however, thus far, replication assays in the absence of assortment assays have given us very little new information beyond what our current plaque assay tells us (aside from the possibility that a fully sorted, fully replicated virus is made that is non-infectious for unknown reasons). For these reasons, we believe identifying the step in the packaging process that is defective in the mutants presented in this manuscript is, though important and ongoing, beyond the scope of this study. Additionally, as already stated, we have now added new data on the one UTR-only mutant we could rescue (18/S1/40) showing there is no evidence of defects in early steps in the life cycle (entry/transcription/translation), but the virus is defective in a late step in the life cycle (see response to 1h above).**

3(d) Experimental proof or an explanation of how sufficient protein is produced for nonrecoverable viruses would be helpful.

**Response: We have included RT-qPCR and immunoblots to show that RNA and protein expression from mutant plasmid constructs are not significantly different to the expression from the corresponding WT or 200/S gene/200 constructs, and therefore sufficient protein is likely present for the constructs that were unrecoverable. To provide further confidence, we have also performed transcomplementation assays for a subset of constructs (eg. S2, S1 UTR mutants). In that assay, we found that even with additonal protein expression, no viruses were recovered (Fig 3E/F and Fig. 10) suggesting that the defect is likely at a step that is independent of how much protein is present. Finally, in the revision, we have repeated immunoblot assays on a subset of our mutants to show that protein expression remains similar in the presence of the other plasmids present in the RG assay (Fig. 3H). While these experiments do not prove that enough protein is being provided, they do provide strong evidence that there is no difference in protein expression levels between viruses that are rescued and viruses that are not, which strongly suggest protein is present in sufficient levels to rescue virus.**

**Additional information:**

1. **We have added “Investigation” in the author contributions of Cathy L. Miller (Line 19).**
2. **Added Jake Peterson and Gage Haines in the acknowledgment section (Lines 838-845).**
3. **Added and/or modified protocol description in Lines 814-825, 793-805, 746-752, 743, 735, 708-713, 659.**
4. **Added and/or modified figure legends in lines 1111-1112, 1101-1102, 1092, 1065, 1051-1053, 1021-1040, 1013, 1019-1020**
5. **Added description of new results corresponding to Figure 3 addressing reviewers concerns (Lines 256-299.**
6. **Added line 219-220,**
7. **Additonal changes in reference numbers, abbrevations, spellings and grammar are tracked throughout the redlined document.**

**References referred to in the response to review.**

1.	Xu Z, Mathews DH. Multilign: an algorithm to predict secondary structures conserved in multiple RNA sequences. Bioinformatics. 2011;27(5):626-32.

2.	Roner MR, Steele BG. Features of the mammalian orthoreovirus 3 Dearing l1 single-stranded RNA that direct packaging and serotype restriction. J Gen Virol. 2007;88(Pt 12):3401-12.

3.	Roner MR, Steele BG. Localizing the reovirus packaging signals using an engineered m1 and s2 ssRNA. Virology. 2007;358(1):89-97.

4.	Roner MR, Roehr J. The 3' sequences required for incorporation of an engineered ssRNA into the Reovirus genome. Virology Journal. 2006;3(1):1.

5.	Roner MR, Joklik WK. Reovirus reverse genetics: Incorporation of the CAT gene into the reovirus genome. Proceedings of the National Academy of Sciences of the United States of America. 2001;98(14):8036-41.

6.	Demidenko AA, Blattman JN, Blattman NN, Greenberg PD, Nibert ML. Engineering recombinant reoviruses with tandem repeats and a tetravirus 2A-like element for exogenous polypeptide expression. Proceedings of the National Academy of Sciences of the United States of America. 2013;110(20):E1867-76.
